# Midnight in a Prison

**Published:** 1882-01

**Authors:** 


### MIDNIGHT IN A PRISON.

There is something very solemn in a large convict prison at midnight. A faint sound of healthy slumber comes from the cells where the convicts sleep. Perhaps there are a thousand, perhaps only five hundred, undergoing punishment; but whatever may be the number, one is conscious that nowhere else save in a convict prison could so many human beings sleep with so little to interrupt the sense of calm repose. In the same number of persons taken from the ordinary world, there would be slight sounds arising from nightmare following on indigestion—perhaps from some reminiscence troubling the conscience on the question whether the strong steps taken for payment of that bill were not in the circumstances slightly harsh, or some other disturbing recollection; there might also be uneasy thoughts and dreams creative of restlessness. None of these troubles disturb the sleep of the habitual criminal. This is not because his conscience lies easy on him, but because he does not possess the article known to the rest of the world as a conscience. Hence he neither enjoys the satisfaction of its healthy and genial condition nor the troubles attending on its inflictions, and it is with him essentially that the "Prayer for Indifference," by Greville, as it may be found in the old "Elegant Extracts," is granted.—*Blackwood's Magazine.*

NURSERY RHYME—OF THE POLYGLOT CLASS.

Patti cake, Patti cake, Franchi man !
" So I do, messieurs, *comme vite* as I can."
*Roulez et tournez et marquez :* " with care,"
*Et posez au publique à* TEN DOLLARS A CHAIR !
—*Puck.*

" WHAT rascal's got my knife ?" exclaimed Fenderson. "Oh, I've got it myself," he added, after another search of his pockets. And the boys said : "That's right, Fenderson, own up like a little man." Fenderson couldn't for the life of him tell what they were driving at.—*Boston Transcript.*

